# Supplementary material for: The case for extended lifespan in cooperatively breeding mammals: a re-appraisal
Source: PeerJ. 2020 May 19;8:e9214. doi: 10.7717/peerj.9214 (PMC7243813; doi:10.7717/peerj.9214)
Supplement: Supplemental Information 1 — Models were fitted using a phylogenetic linear mixed effects modelling framework to dataset excluding the cape mole-rat (Georychus capensis), and all arboreal or semi-arboreal taxa. Estimate refers to the mean of the posterior distribution from models fitted to 100 different mammalian trees. Terms where the 95% credible intervals which do not overlap zero are deemed biologically significant. Significant model terms are highlighted in bold. Note that the reference category refers to non-cooperatively breeding, ground-dwelling species. [file peerj-08-9214-s001.docx]

**SUPPLEMENTARY INFORMATION**

**Thorley (2020) The case for extended lifespan in cooperatively breeding mammals: a re-appraisal**

**Table S1. Phylogenetic analyses of maximum lifespan across terrestrial mammals, fitted to different subsets of the global dataset. Models were fitted using a phylogenetic linear mixed effects modelling framework to dataset excluding the cape mole-rat (*Georychus capensis*), and all arboreal or semi-arboreal taxa. Estimate refers to the mean of the posterior distribution from models fitted to 100 different mammalian trees. Terms where the 95% credible intervals which do not overlap zero are deemed biologically significant. Significant model terms are highlighted in bold. Note that the reference category refers to non-cooperatively breeding, ground-dwelling species.**

|  | **Global dataset without the cape mole-rat** | **Global dataset excluding arboreal/semi-arboreal species** |
| --- | --- | --- |
| **Model Term** | Mean estimate (95% CI) | Mean estimate (95% CI) |
| Intercept | 2.724 (2.127 – 3.324) | 2.804 (2.176 – 3.434) |
| Adult body mass | **0.332 (0.274 – 0.390)** | **0.352 (0.274 – 0.432)** |
| Cooperative breeding status: cooperative | 0.064 (-0.022 – 0.149) | 0.088 (-0.016 – 0.192) |
| Fossoriality: subterranean | **0.349 (0.093 – 0.149)** | **0.335 (0.069 – 0.599)** |
| Lifestyle: semi-arboreal | 0.083 (-0.009 – 0.175) | – |
| Lifestyle: arboreal | **0.130 (0.024 – 0.235)** | – |
| Sample size: medium | **0.127 (0.089 – 0.166)** | **0.125 (0.076 – 0.173)** |
| Sample size: large | **0.207 (0.155 – 0.259)** | **0.201 (0.134 – 0.267)** |
|  |  |  |
| Residual variance | 0.015 (0.010 – 0.021) | 0.015 (0.009 – 0.022) |
| Phylogenetic variance | 0.389 (0.314 – 0.465) | 0.418 (0.320 – 0.516) |
|  |  |  |
| Marginal R2 | 0.258 (0.190 – 0.336) | 0.269 (0.182 – 0.372) |
| Conditional R2 | 0.972 (0.958 – 0.982) | 0.974 (0.957 – 0.985) |
|  |  |  |
| Species number | 718 | 499 |
